# Xinmai 'an extract enhances the efficacy of sildenafil in the treatment of pulmonary arterial hypertension via inhibiting MAPK signalling pathway

**DOI:** 10.1080/13880209.2021.1917629

**Published:** 2021-05-19

**Authors:** Yaolu Zhu, Yabin Sun, Shichang Zhang, Chuyuan Li, Yiwei Zhao, Boxin Zhao, Guofeng Li

**Affiliations:** a Department of Pharmacy, Nanfang Hospital, Southern Medical University, Guangzhou, China; b Rational Medication Evaluation and Drug Delivery Technology Lab, Nanfang Hospital, Southern Medical University, Guangzhou, China; c Modern Chinese Medicine Institute, Hutchison Whampoa Guangzhou Baiyunshan Chinese Medicine Company Limited, Guangzhou, China; d Office of the General Manager, Hutchison Whampoa Guangzhou Baiyunshan Chinese Medicine Company Limited, Guangzhou, China; e Guangdong Key Laboratory of New Drug Screening, Southern Medical University, Guangzhou, China

**Keywords:** Pulmonary artery pressure, oxidative stress, inflammation, combination treatment

## Abstract

**Context:**

Xinmai 'an tablet has been used to improve myocardial blood supply. Recently, some compounds from its formula have shown that they can treat pulmonary arterial hypertension (PAH).

**Objective:**

This study investigates the effects of Xinmai 'an extract (XMA) on PAH and further tests the co-therapeutic enhancement with sildenafil (SIL).

**Materials and methods:**

Pulmonary artery smooth muscle cells were subjected to stimulation with SIL (12.5 μM) and XMA (250 μg/mL) for 48 h. Sprague–Dawley rats were randomly grouped into eight groups (*n* = 8 per group): (I) control group received saline; (II) MCT group received MCT (60 mg/kg); (III) SIL-Low group received MCT + SIL at 10 mg/kg/day; (IV) SIL-high group received MCT + SIL at 30 mg/kg/day; (V) XMA-High group received MCT + XMA at 251.6 mg/kg/day; (VI) SIL (Low)+XMA (Low) group received SIL (10 mg/kg) + XMA at 62.9 mg/kg/day; (VII) SIL (Low)+XMA (Medium) group received SIL (10 mg/kg) + XMA at 125.8 mg/kg/day; (VIII) SIL (Low)+XMA (High) group received SIL (10 mg/kg) + XMA at 251.6 mg/kg/day. Both XMA and SIL were given by gavage and were maintained daily for 2 weeks.

**Results:**

XMA could improve SIL’s efficacy in the treatment of PAH by decreasing cell viability more effectively at non-cytotoxic concentrations (250 μg/mL) and reducing Right Ventricular Systolic Pressure (RVSP) in PAH rat. Potential mechanisms might at least in part be through activating the MAPK signalling pathway.

**Discussion and conclusions:**

The combination of XMA and SIL can improve the efficacy of pulmonary hypertension and reduce the dosage of SIL.

## Introduction

Pulmonary hypertension refers to a hemodynamic and pathophysiological state in which the increase of pulmonary artery pressure exceeds a certain threshold, which can lead to right heart failure. It is a common and frequently-occurring disease with a high disability rate and fatality rate, which should arouse people’s great attention (Voelkel et al. 2012; Zangiabadi et al. 2014). The primary treatments for pulmonary arterial hypertension (PAH) include epoprostenol and derivatives, endothelin receptor antagonists, calcium channel blockers, and recently researchers have focussed on phosphodiesterase type 5 enzyme (PDE-5) inhibitors such as sildenafil (SIL), which mainly focus on expanding blood vessels selectively, weakening anti-proliferation ability of PASMCs (Montani et al. 2014). These drugs can improve the quality of life of patients with pulmonary hypertension to a certain extent. However, none of the aforementioned drugs, including SIL, can completely reverse the development of the disease, and there are many adverse reactions (Hao et al. 2020). Therefore, there is an urgent need for more new methods to treat PAH.

The pathogenesis of pulmonary hypertension involves a complex process and is affected by many factors, including the excessive proliferation of PASMCs, inflammation activation, oxidative stress and deregulated immunity (Pietra et al. 2004; Huertas et al. 2014). Among them, the migration and proliferation of PASMCs is the main pathological basis for pulmonary artery remodelling and pulmonary hypertension (Sakao and Tatsumi 2011). Recent studies revealed that the process of pulmonary blood vessel formation and remodelling involves multiple signalling pathways, including the mitogen-activated protein kinase (MAPK) signalling pathway, nuclear factor-κB (NF-κB) signalling pathway, and PI3K/AKT signalling pathway (Li et al. 2014; 2016; Wang et al. 2019). Therefore, direct or combined targeted therapy to inhibit the activation of signal pathways may be an effective way to treat pulmonary hypertension.

Xinmai 'an tablet is a Chinese herbal compound preparation consisting of 6 traditional Chinese medicines such as *Panax ginseng* C. A. Mey (Araliaceae), *Astragalus membranaceus* (Fisch.) Bunge (Leguminosae), *Salvia miltiorrhiza* Bunge (Labiatae), Paeoniae Radix Rubra (Ranunculaceae), Ophiopogonis Radix (Liliaceae) and Borneolum Syntheticum. Xinmai 'an tablet is traditionally for the treatment of cardiovascular disease (Shao 2013). Interestingly, the main components of Xinmai 'an tablets can inhibit endothelin-1 levels, increase NO content, dilate blood vessels, and increase blood flow. Furthermore, according to the results of high-performance liquid chromatography from our previous study (Zhu et al. 2021), the major chemical ingredients of Xinmai 'an extract are catechinic acid, paeoniflorin, ginsenoside Rg_1_, kaempferol, ginsenoside Rg_3_ and Tanshinone IIA. Some of these monomers have been reported to treat pulmonary hypertension, ginsenoside Rg_1_ has been demonstrated to possess a significant role in the inhibition of hypoxia and hypercapnia-induced vasoconstriction by the p38 pathway (Zheng et al. 2016). Paeoniflorin improves pulmonary hypertension by inhibiting endothelial transition to the mesenchymal (Andersen et al. 2019). Kaempferol could alleviate pulmonary hypertension caused by chronic hypoxia (Nan et al. 2018). Tanshinone IIA could improve right ventricular hypertrophy and reduce pulmonary artery smooth muscle thickening (Hu et al. 2017).

Considering the properties of some components of XMA, we hypothesized that it may itself have an effect on PAH, and as one of Chinese Traditional Medicine, it may exert synergism with some clinically used medicines such as SIL which can alleviate pulmonary hypertension, but the price is higher and has more side effects. Therefore, in this work, we investigated the role and mechanism of XMA alone or in combination with SIL in the abnormal proliferation of PASMCs. Furthermore, we also studied the preventive and therapeutic effects of XMA and SIL in pulmonary hypertension rats. The experimental study here revealed the key role of XMA in the treatment of PAH, and the combination with SIL may have a dual effect and provide better survival than pulmonary vasodilators alone.

## Materials and methods

### Materials

Xinmai 'an extract was supported from Hutchison Whampoa Guangzhou Baiyunshan Chinese Medicine Company Limited Modern Chinese Medicine Institute. (Guangzhou, China, batch number: 190701). Sildenafil and monocrotaline were obtained from Shanghai Yuanye Bio-technology Co. FITC-Annexin V and propidium iodide (PI) were purchased from BD Biosciences. Antibodies against p38, p-p38, ERK, p-ERK, JNK, p-JNK, caspase 3, cleaved-caspase 3, α-SMA were obtained from Cell Signalling Technology (CST). Antibodies against Bcl-2, Bax, MMP2, MMP9 were obtained from Abclonal. Recombinant Human Platelet-Derived Growth Factor-BB (PDGF-BB) was obtained from Dakewe Biotech Co., Ltd.

### Extract preparation

Powdered raw material of *Panax ginseng*, *Astragalus membranaceus*, *Salvia miltiorrhiza*, *Paeoniae* Radix Rubra*, Ophiopogonis* Radix and Borneolum Syntheticum were crushed into a coarse powder and extracted by a refluxing method for 2 h, the filtrates were collected and concentrated into the extract.

### Cell culture

Primary pulmonary artery smooth muscle cells (PASMCs) were purchased from OTWO BIOTECH INC (rat, HTX2164), cell culture media and supplements were products of GIBCO-BRL. The cells were cultured in DMEM containing 10% FBS at 37 °C in a humidified atmosphere of 95% air and 5% CO_2_. The cells from passages 4–8 were used in all experiments.

### Cell viability and proliferation assay

Cell viability and proliferation were measured by the cell counting kit-8 assay. PASMCs were seeded in 1 × 10^4^ cells per well in 96-well plates for 24 h and incubated with serum-deprived in 1% FBS for 24 h. For the viability assay, the cells were treated with various concentrations (0, 2.5, 5, 10, 20, and 40 ng/mL) of PDGF-BB for 24, 48, and 72 h to determine the dosage of PDGF-BB. Next, the cells were treated with various concentrations (0, 6.25, 12.5, 25, and 50 μM) of SIL or various concentrations (0, 125, 250, 500 and 1000 μg/mL) of XMA for 48 h to determine the toxicity of drugs. For proliferation assay, PASMCs were pre-treated with 40 ng/mL PDGF-BB and then subjected to stimulation with various concentrations of SIL or various concentrations of XMA which were under the safe dosage for 48 h to determine the effect of SIL or XMA to inhibit the proliferation of cells. Next, in order to study the synergistic effect of SIL and XMA to inhibit the proliferation of cells, the cells were pre-treated with 40 ng/mL PDGF-BB and then subjected to stimulation with SIL (12.5 μM) and XMA (250 μg/mL) for 48 h. After the stimulation, CCK8 was added to each well for an additional 2 h. The absorbance was measured at 450 nm by a SpectraMax M5 Multi-function microplate reader (Shanghai Enzyme-Linked Biotechnology Co., Ltd).

### Apoptosis assays

Annexin V + PI staining was used to detect the apoptosis of PASMCs by flow cytometry. The mitochondrial membrane potential was examined using Mitochondrial Membrane Potential Assay Kit (JC-1) (Solarbio, China) according to the manufacturer’s instructions.

### Scratch wound assay and transwell migration assay

For wound healing assay, when the density of PASMCs reached 90–100%, the cells were starved for 24 h, and then scratched with a 100 μL pipette tip. After incubating with PDGF-BB with/without SIL or XMA for 48 h, the number of cell migration was calculated under a phase Leica 2500 microscope. For the transwell migration chamber assay, PDGF-BB with/without SIL or XMA were added to the lower chamber when PASMCs (5 × 10^4^ cells in 100 μL serum-free medium) were placed in the upper chamber. Forty-eight hours later, the unmigrated cells were gently removed from the top, and the cells migrated to the submembrane were fixed with 4% paraformaldehyde, stained with 5% crystal violet, and counted under the Leica 2500 microscope.

### Determination of oxidative stress

Intracellular Reactive Oxygen Species changes were determined by measuring the oxidative transformation of 2′,7′-dichlorofluorescein diacetate (DCFH-DA) to fluorescent dichlorofluorescein (DCF) using Reactive Oxygen Species Assay Kit (Solarbio, China). Briefly, cells were inoculated in 6-well plates for 24 h and treated with drugs of different concentrations for 48 h. After that, cells were collected and washed with PBS 3 times. Finally, DCFH-DA was added again and incubated at 37 °C for 20 min. Then the fluorescence was detected by an inverted fluorescence microscope according to the manufacturer’s instructions. Malondialdehyde content in PASMCs was detected by Lipid Peroxidation MDA Assay Kit (Beyotime, China) according to the manufacturer’s protocol and measured at 532 nm by a microplate reader.

### RT-qPCR

RNA derived from PASMCs was extracted using RNAiso Plus reagent (Takara Bio Inc., Shiga, Japan). Reverse transcription was conducted with 2000 ng of total RNA with SYBR Premix Ex Taq kit (Takara Bio Inc., Dalian, China). Quantitative RCR was performed using QuantStudio 5 Real-Time PCR system (Applied Biosystems). GAPDH was selected as a housekeeping gene. PCR primers are listed in Table 1.

**Table 1.** The primer sequences of targeted RNA.

Gene primer	Species		Sequence (5′ to 3′)
IL-1β	Rat	Forward	CCTGGGCTGTCCTGATGAGAGTCCAC
		Reverse	GGGAAAGACACAGGTA
IL-6	Rat	Forward	CTGGTCTTCTGGAGTTCCGTGCCACT
		Reverse	CCTTCTGTGACTCT
TNF-α	Rat	Forward	ATCCGAGATGTGGAACTGACTGATGA
		Reverse	GAGGGAGCCCAT
GAPDH	Rat	Forward	CAATGTGTCCGTCGTGGATCTGTCCT
		Reverse	CAGTGTAGCCCAAGATG

### Animals

**Figure 1.**
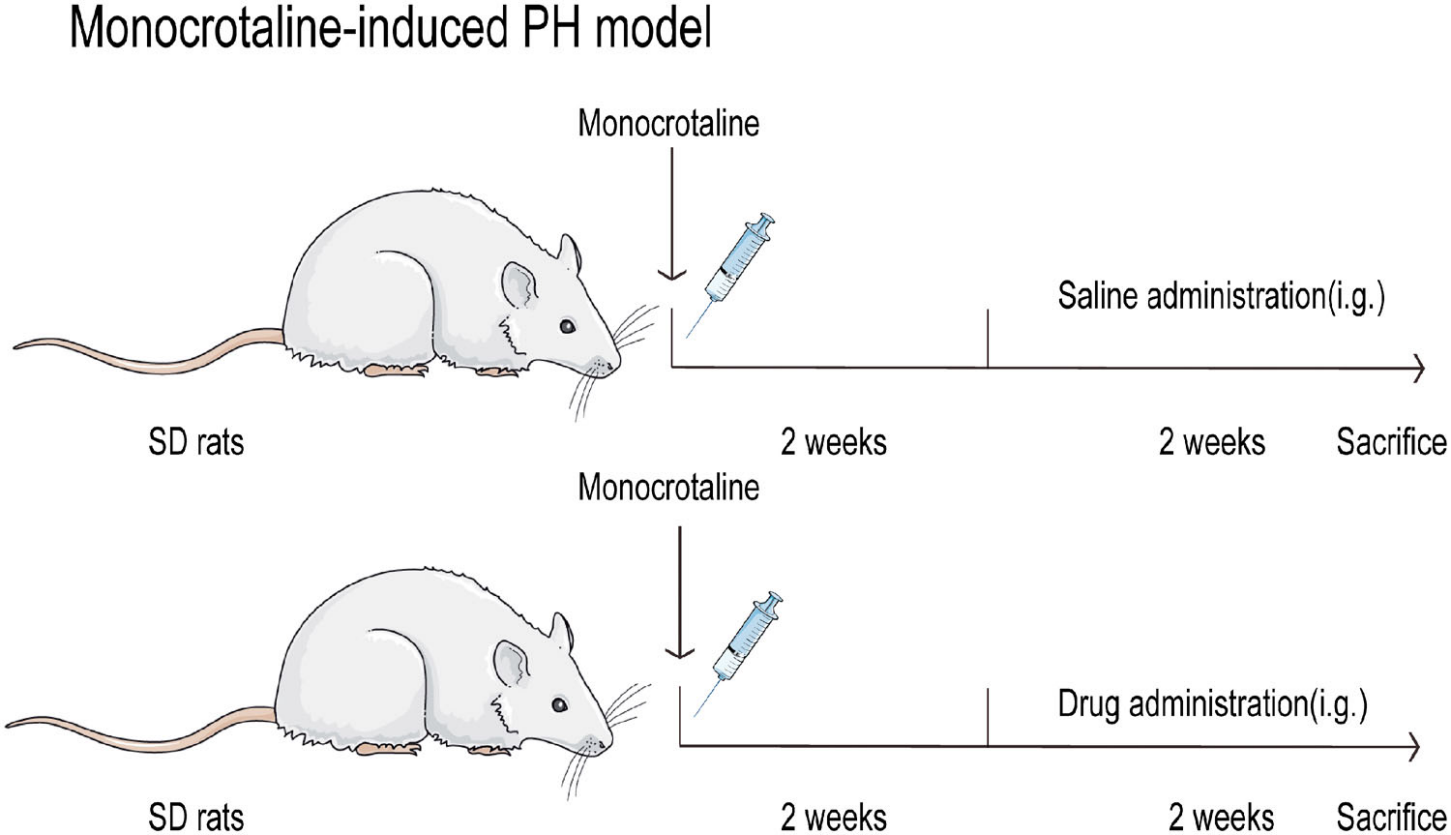
A diagram of the experiment design in rats.

Male Sprague–Dawley rats (220–250 g), provided by the Animal Centre of Southern Medical University, permit number for rats is 440021000025211. The feeding environment of the rats: humidity 50–60%, temperature 22–24 °C, alternating light and dark for 12 h, free eating and drinking. The experiment started after one week of adaptive feeding. All operations during the research were in compliance with the experimental animal management method issued by the Ministry of Science and Technology of China and approved by the Animal Ethics Committee of Southern Medical University. Animals were randomly grouped into eight groups (*n* = 8 per group): (I) control group received saline; (II) MCT group received MCT (60 mg/kg); (III) SIL-Low group received MCT + SIL at 10 mg/kg/day; (IV) SIL-high group received MCT + SIL at 30 mg/kg/day; (V) XMA-High group received MCT + XMA at 251.6 mg/kg/day; (VI) SIL (Low)+XMA (Low) group received SIL (10 mg/kg) + XMA at 62.9 mg/kg/day; (VII) SIL (Low)+XMA (Medium) group received SIL (10 mg/kg) + XMA at 125.8 mg/kg/day; (VIII) SIL (Low)+XMA (High) group received SIL (10 mg/kg) + XMA at 251.6 mg/kg/day. Pulmonary hypertension was induced by subcutaneous injection of MCT into the back of rats. The drug treatments started 2 weeks after the MCT injection and were maintained daily for 2 weeks. Both XMA and SIL were given by gavage. All experimental animals were weighed every other day, and the dosage was adjusted according to their body weight. The dosage regimen is shown in Figure 1.

### Hemodynamic evaluation

Twenty-eight days later, the rats were weighed (body weight, BW) and anaesthetized by intraperitoneal injection of sodium pentobarbital (40 mg/kg). As described in the previous study (Li et al. 2016), a catheter was inserted into the pulmonary artery of the rat and the RV systolic pressure (RVSP) was measured according to the waveform shown on the biological function laboratory system (BL-420, Tai Meng Technology Co., Ltd., Chengdu, China). After RVSP was measured, the hearts were removed from the anaesthetized rats and placed in cold normal saline. The right ventricle and left ventricle, as well as the ventricular septum, were then separated and weighed, and the right cardiac hypertrophy index (RVHI) was calculated by determining the weight ratio of the right ventricle (RV) to the left ventricle (LV) and the ventricular septum (S) (RV/[LV + S]). The lung and heart tissues were collected for western blotting (stored at −80 °C) and histological analysis (fixed in 4% paraformaldehyde). Blood drawn from animals was centrifuged at 3000 rpm at 4 °C for 10 min and then stored at −80 °C.

### Histology measurements and immunofluorescence

Briefly, lung tissue sections were stained with haematoxylin-eosin staining to observe the morphology of blood vessels, NDP.view2 software was used to measure the pulmonary arterial media wall thickness. α-SMA immunofluorescence was used to analyze the muscular level of the lung. RV fibrosis was assessed by Masson’s trichrome staining.

### Western blot

As described previously (Wang et al. 2018), protein extracted from tissues and cells was separated via 10% or 12% SDS-PAGE and transferred onto PVDF membranes (Bio-Rad Laboratories, USA). Afterwards, the PVDF membranes were sealed with 5% skimmed milk at room temperature for 1 h, and incubated overnight with the primary antibodies at 4 °C. Then, PVDF membranes were washed by TBST for 30 min and incubated with HRP-conjugated second antibody for 1 h. After washing PVDF membrane with TBST for 30 min again, chemiluminescence (ECL, Millipore, Bedford, MA, USA) was employed to label the protein band. The obtained pictures were quantitatively analyzed with Image Lab software to determine the grey value. With GAPDH or β-actin as an internal reference, the bands of each objective were standardized, and the differences between the groups were compared.

### Statistical analysis

GraphPad Prism software version 8 was used for statistical analysis, and all experiments with at least three replicates were expressed as mean ± SEM. The two groups were analyzed by Student’s *t*-test, or multiple groups were analyzed by one-way ANOVA with Tukey posthoc test. *p*-Values < 0.05 were considered statistically significant.

## Results

### Effects of PDGF-BB, SIL and XMA on proliferation of PASMCs

**Figure 2.**
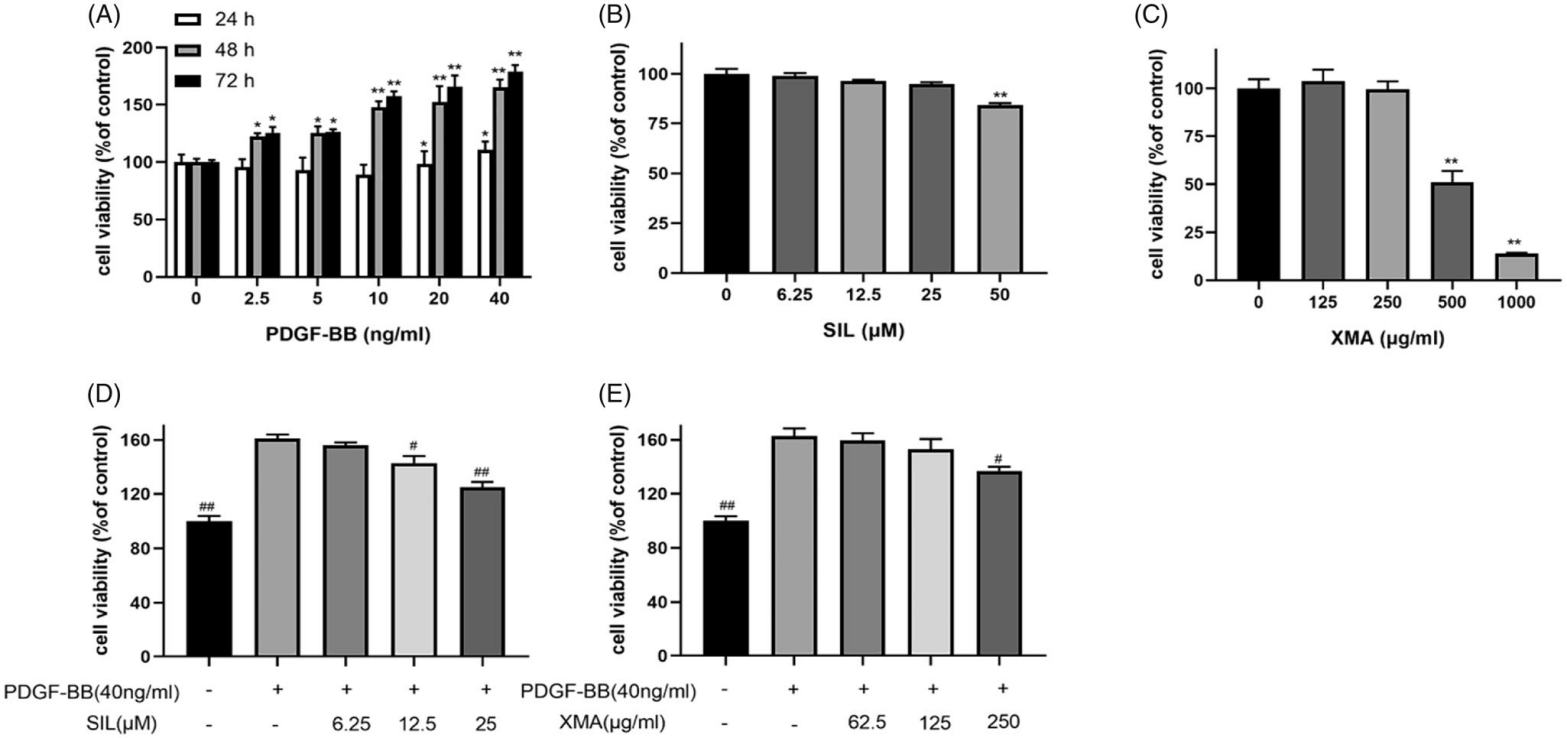
Effects of PDGF-BB, SIL and XMA on proliferation of PASMCs. (A) Effects of PDGF-BB on proliferation of PASMCs with different concentrations and different times were assessed by CCK8 assay. (B, C) The cytotoxicity of SIL and XMA on cells with different concentrations for 48 h. (D, E) Effects of SIL and XMA on proliferation of 40 ng/mL PDGF-BB induced PASMCs. Data are presented as the mean ± SEM. (*n* = 3). *p < 0.05, **p < 0.01 vs. control. #*p* < 0.05, ##*p* < 0.01 vs. PDGF-BB group.

Firstly, we used the CCK8 assay to detect effects of PDGF-BB on PASMCs viability. As shown in (Figure 2(A)), 40 ng/mL PDGF-BB significantly increased cell activity at 48 h. Then to detect effects of SIL and XMA on PASMCs viability, SIL (6.25–25 μM) (Figure 2(B)) and XMA (125–250 μg/mL) (Figure 2(C)) had no toxic effects on PASMCs after 48 h of exposure. To investigate SIL and XMA in PDGF-BB induced PASMCs proliferation, exposure of cells to PDGF-BB with various concentration of SIL (6.25, 12.5 and 25 μM) (Figure 2(D)) or with XMA (62.5, 125 and 250 μg/mL) for 48 h (Figure 2(E)) was detected by the CCK8 assay. Compared with PDGF-BB group, the number of cells in the SIL and XMA groups was reduced. These results indicated that SIL and XMA can both weaken the proliferation of cells.

### Effects of SIL and XMA administered separately or in combination on proliferation and migration of PASMCs

**Figure 3.**
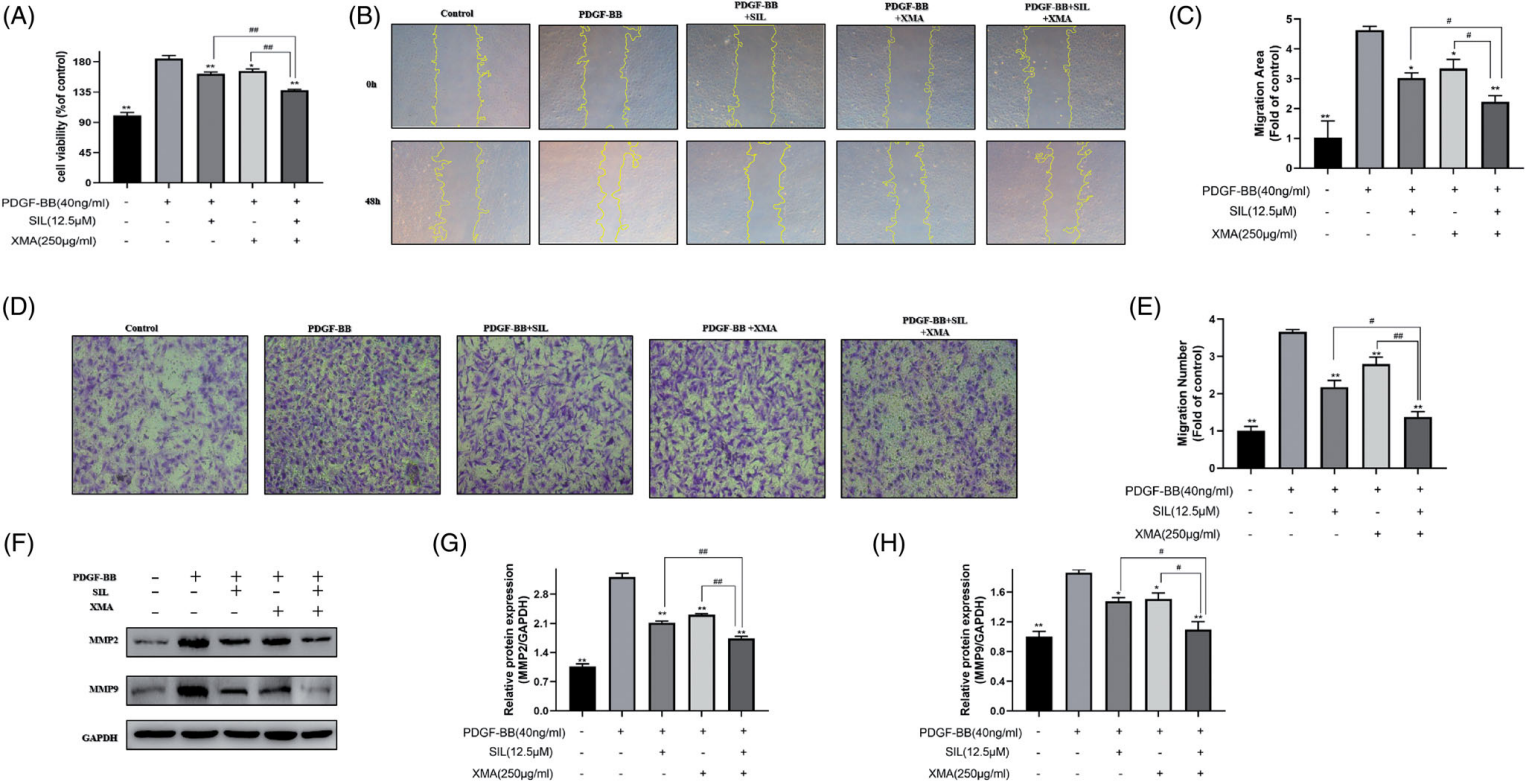
Effects of SIL and XMA administered separately or in combination on proliferation and migration of PASMCs. (A) Effects of SIL in combination with XMA on proliferation of cells. (B, C) Effects of SIL in combination with XMA on migration of cells by wound healing. (D, E) Effects of SIL in combination with XMA on migration of cells by transwell migrations. (F–H) The expression of MMP2 and MMP9 were analyzed with western blotting and the protein expression ratio of MMP2 and MMP9 to GAPDH was evaluated. Data are presented as the mean ± SEM. (*n* = 3). *p < 0.05, **p < 0.01 vs. PDGF-BB group; #*p* < 0.05, ##*p* < 0.01 vs. SIL + XMA group.

When cells were treated with the combination treatment of SIL and XMA which comprised a low effective concentration of SIL (12.5 μΜ) and XMA (250 μg/mL) for 48 h, the proliferation of cells was decreased effectively (Figure 3(A)). To test whether XMA could increase the effectiveness of SIL in reducing 40 ng/mL PDGF-BB-induced PASMCs proliferation and migration, cells were treated with SIL and XMA separately or in combination for 48 h, and wound healing and transwell migration was performed. Treated with XMA, cell migration was inhibited. And when the cells were exposed to XMA that combined with SIL, the cell migration rate was significantly reduced (Figure 3(B–E)). To further elucidate the mechanism of inhibitory effects on migration, we measured the activity and expression of MMP2 and MMP9, which can break down most components of the extracellular matrix and are positively correlated with the degree of migration (Xiao et al. 2018). The increasing expression of PDGF-BB stimulated MMP2 and MMP9 was inhibited by SIL and XMA treatment (Figure 3(F–H)).

### Effects of SIL and XMA administered separately or in combination on apoptosis of PASMCs

**Figure 4.**
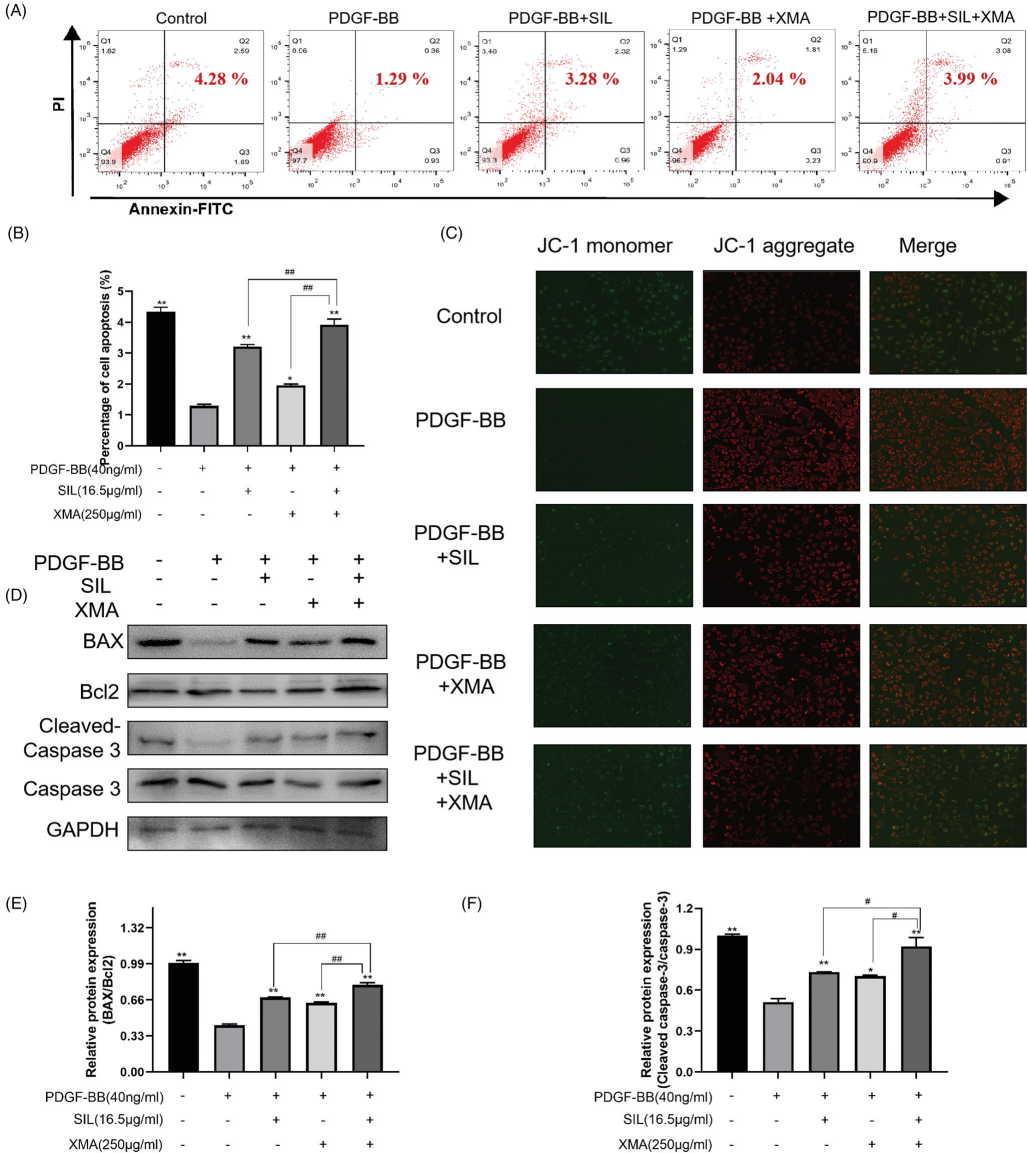
Effects of SIL and XMA administered separately or in combination on apoptosis of PASMCs. (A, B) The apoptosis of PASMCs was detected by flow cytometry by staining with Annexin V + PI. (C) JC-1 staining. (D–F) The expression of BAX, Bcl2, Cleaved-Caspase 3 and Caspase were analyzed with western blotting and the protein expression ratio of BAX/Bcl2, Cleaved-Caspase 3/Caspase was evaluated. Data are presented as the mean ± SEM. (*n* = 3). *p < 0.05, **p < 0.01 vs. PDGF-BB group; #*p* < 0.05, ##*p* < 0.01 vs. SIL + XMA group.

We investigated whether the combination of SIL and XMA could exhibit an inhibition potential of the proliferation of PASMCs effectively, and we further investigated whether the combination of SIL and XMA could further promote PASMCs apoptosis by using flow cytometry, JC-1 staining, and western blot. As indicated in Figure 4(A,B). The number of apoptotic cells was decreased by 40 ng/mL PDGF-BB stimulation for 24 h. However, SIL (12.5 μΜ) and XMA (250 μg/mL) treatment increased the number of apoptotic cells. At the same time, when the cells were exposed to XMA that combined with SIL, the number of apoptotic cells was increased. The decrease of mitochondrial membrane potential is a landmark event in the early stage of apoptosis. The transition of JC-1 from red fluorescence to green fluorescence can easily detect the drop of cell membrane points, and the transition from red fluorescence to green fluorescence of JC-1 can also be used as an early detection indicator of apoptosis (Salimiaghdam et al. 2020). The results from JC-1 staining and western blot were consistent with flow cytometry (Figure 4(C–F)). These results indicated that SIL combining with XMA could increase the percentage of apoptotic cells.

### Effects of SIL and XMA administered separately or in combination on oxidative stress levels and inflammatory response

**Figure 5.**
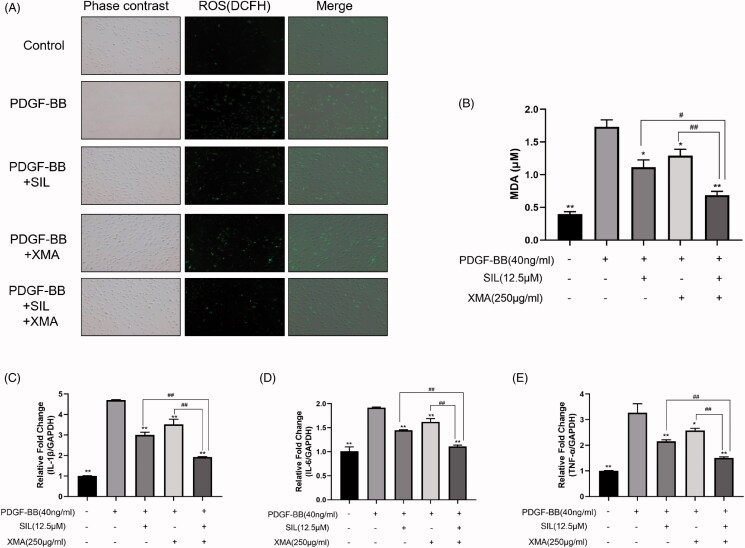
Effects of SIL and XMA administered separately or in combination on oxidative stress levels. (A) The level of ROS in PASMCs. (B) The level of MDA in PASMCs. (C) The level of IL-1β in PASMCs. (D)The level of IL-6 in PASMCs. (E) The level of TNF-α in PASMCs. Data are presented as the mean ± SEM. (*n* = 3). *p < 0.05, **p < 0.01 vs. PDGF-BB group; #*p* < 0.05, ##*p* < 0.01 vs. SIL + XMA group.

Oxidative stress and inflammatory response are major factors in the progression of PAH. With the development of PAH, circulating monocytes accumulate in pulmonary arterioles and begin to produce ROS, inducing cell proliferation and fibrosis of the pulmonary artery and RV (Daneva et al. 2019). And given that therapeutics targeting inflammation could weaken the development of PAH (Shen et al. 2019), to clarify whether SIL in combination with XMA can treat pulmonary hypertension by reducing oxidative stress levels and inflammatory response, we detected the contents of ROS, MDA and inflammatory factors *in vitro*. As shown in Figure 5(A,B), 40 ng/mL PDGF-BB increased ROS and MDA generation in PASMCs, which were abolished by SIL (12.5 μΜ) and XMA (250 μg/mL) treatment. Meanwhile, when the cells were exposed to XMA that combined with SIL, the level of ROS and MDA was obviously decreased. Similarly, the level of IL-1β, IL-6, TNF-α were down-regulated in the combined group which was compared with SIL or XMA only administered group (Figure 5(C–E)).

### Effects of SIL and XMA administered separately or in combination on RVSP and RV remodelling in rats with MCT-induced PH rats

**Figure 6.**
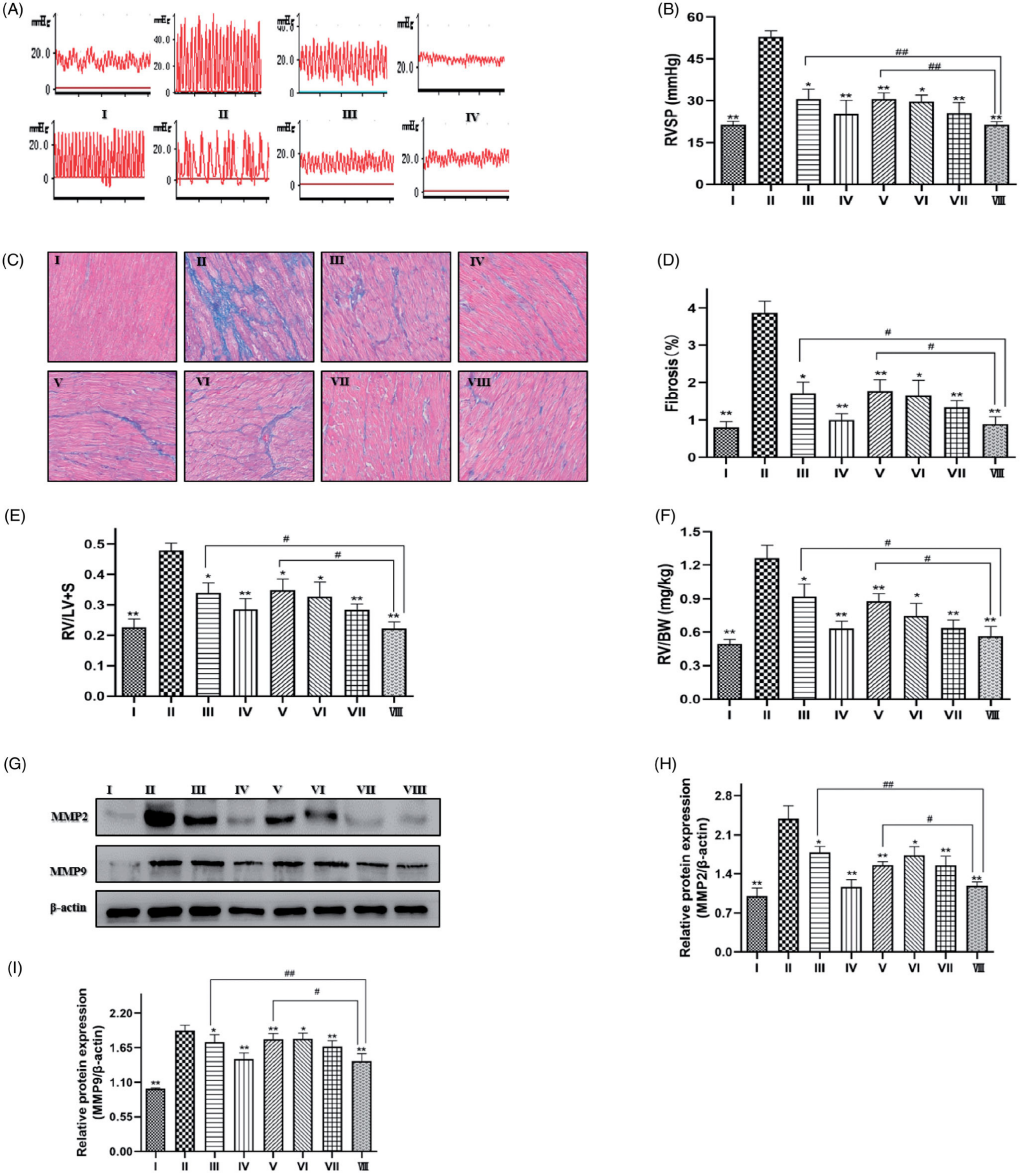
Effects of SIL and XMA administered separately or in combination on RVSP and RV remodelling in rats with MCT-induced PH rats. (A) Representative pictures of RVSP waves in each group. (B) RVSP. (C) Representative photomicrographs from heart tissues with Masson staining. (D) Mean cross section area (CSA) of cardiomyocytes was evaluated based on the results of Masson. (E) RV/LV + S ratio. (F) RV/body weight ratio. (G–I) The expression of MMP2 and MMP9 were analyzed with western blotting and the protein expression ratio of MMP2, MMP9 to β-actin was evaluated. Group I:control group received saline; Group II: MCT group received MCT(60 mg/kg); Group III: SIL-Low group received MCT + SIL at 10 mg/kg/day; Group IV: SIL-high group received MCT + SIL at 30 mg/kg/day; Group V: XMA-High group received MCT + XMA at 62.9 mg/kg/day; Group VI: SIL(Low)+XMA(Low) group received SIL(10 mg/kg) + XMA at 125.8 mg/kg/day; Group VII: SIL(Low)+XMA(Medium) group received SIL(10 mg/kg) + XMA at 251.6 mg/kg/day; Group VIII: SIL(Low)+XMA(High) group received SIL(10 mg/kg) + XMA at 251.6 mg/kg/day. Data are presented as the mean ± SEM. (*n* = 6–8). *p < 0.05, **p < 0.01 vs. Group II; #*p* < 0.05, ##*p* < 0.01 vs. Group VIII.

To further verify the efficacy of the drugs, we conducted experiments in rats. After 28 days, the RVSP of rats in the model group was significantly higher than that of rats in the normal group, but RVSP was reduced when treated with low doses of SIL or high doses of XMA. Combined with a low dose of SIL, RVSP was dramatically decreased with XMA treatment, when the dose of XMA was at its maximum, the result was similar to the level of high dose of sildenafil (Figure 6(A,B)). RV fibrosis and RVHI were calculated to evaluate the effect of SIL or XMA on RV remodelling. The typical pathological changes of Masson staining are shown in Figure 6(C). Statistical analysis shows that the combination of SIL and XMA effectively alleviated MCT-induced increments in RV fibrosis (Figure 6(D)) and RVHI (Figure 6(E,F)). The results from western blot were consistent with Masson staining (Figure 6(G,H)). Together, these results suggest that SIL combining with XMA could prevent MCT-induced pulmonary hypertension and right ventricle dysfunction effectively.

### Effects of SIL and XMA administered separately or in combination on pulmonary arterial remodelling in rats with MCT-induced PH rats

**Figure 7.**
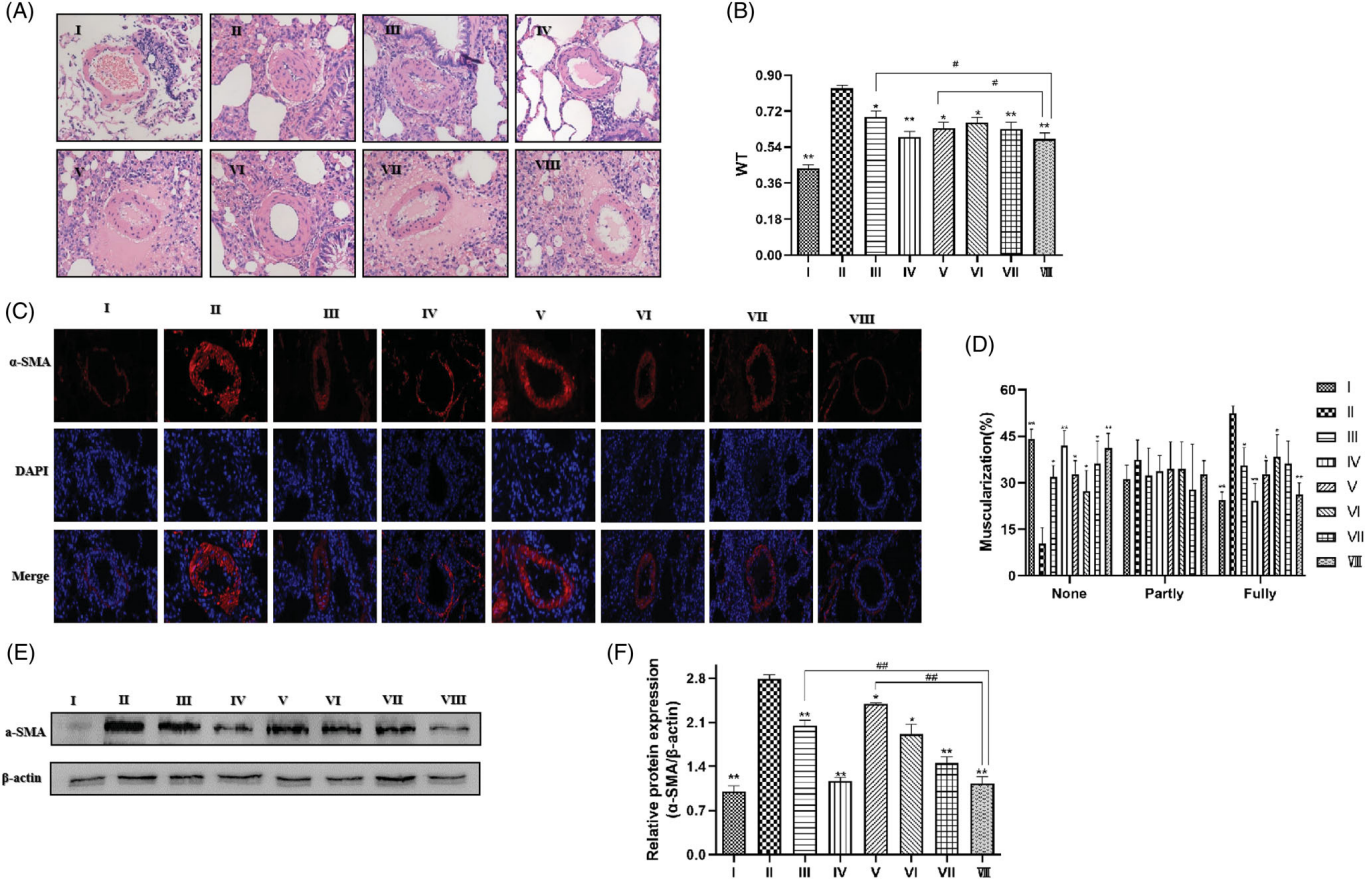
Effects of SIL and XMA administered separately or in combination on pulmonary arterial remodelling in rats with MCT-induced PH rats. (A) HE staining showing representative micrographs of pulmonary artery remodelling. (B) Quantitative analysis of the percentage of medial arteriole thickness. (C) A representative image of the immunohistochemical staining for alpha smooth muscle actin (α-SMA) antibody. (D) The proportion of muscularized small pulmonary arteries. (E, F) The expression of α-SMA were analyzed by western blotting and the protein expression ratio of α-SMA to β-actin was evaluated. Group I:control group received saline; Group II: MCT group received MCT(60 mg/kg); Group III: SIL-Low group received MCT + SIL at 10 mg/kg/day; Group IV: SIL-high group received MCT + SIL at 30 mg/kg/day; Group V: XMA-High group received MCT + XMA at 62.9 mg/kg/day; Group VI: SIL(Low) +XMA (Low) group received SIL(10 mg/kg) + XMA at 125.8 mg/kg/day; Group VII: SIL(Low)+XMA(Medium) group received SIL(10 mg/kg) + XMA at 251.6 mg/kg/day; Group VIII: SIL(Low)+XMA(High) group received SIL(10 mg/kg) + XMA at 251.6 mg/kg/day. Data are presented as the mean ± SEM. (*n* = 6–8). *p < 0.05, **p < 0.01 vs. Group II; #*p* < 0.05, ##*p* < 0.01 vs. Group VIII.

To assess the pulmonary arterial remodelling, we measured the inner wall thickness of the small pulmonary artery (external diameters of 50–100 μm) by haematoxylin and eosin staining, immunohistochemical staining against α-SMA and western blot. As indicated in Figure 7(A,B), PAWT in model rats increased markedly compared to those in normal rats, but PAWT decreased less when rats were treated with a low dose of SIL or XMA (251.6 mg/kg/day). Combined with a low dose of XMA, PAWT was markedly reduced with SIL treatment, similar to the level of high dose of SIL. Immunohistochemical staining against α-SMA was performed to evaluate muscularization in small pulmonary vessels (Figure 7(C)), compared with the control group, MCT stimulation resulted in a significant decrease in the proportion of nonmuscular pulmonary artery and an increase in the fully muscular pulmonary artery, which was attenuated by low doses of SIL or XMA, but remarkably attenuated with XMA treatment in a dose-dependent manner, similar to the level of high dose of SIL (Figure 7(D,E)). The results from western blots were consistent with immunohistochemical staining (Figure 7(F,G)). Together, these results demonstrate that SIL combining with XMA could prevent MCT-induced pulmonary arterial remodelling effectively.

### Effects of SIL and XMA administered separately or in combination could inhibit MAPK signalling pathway *in vitro* and *in vivo*

**Figure 8.**
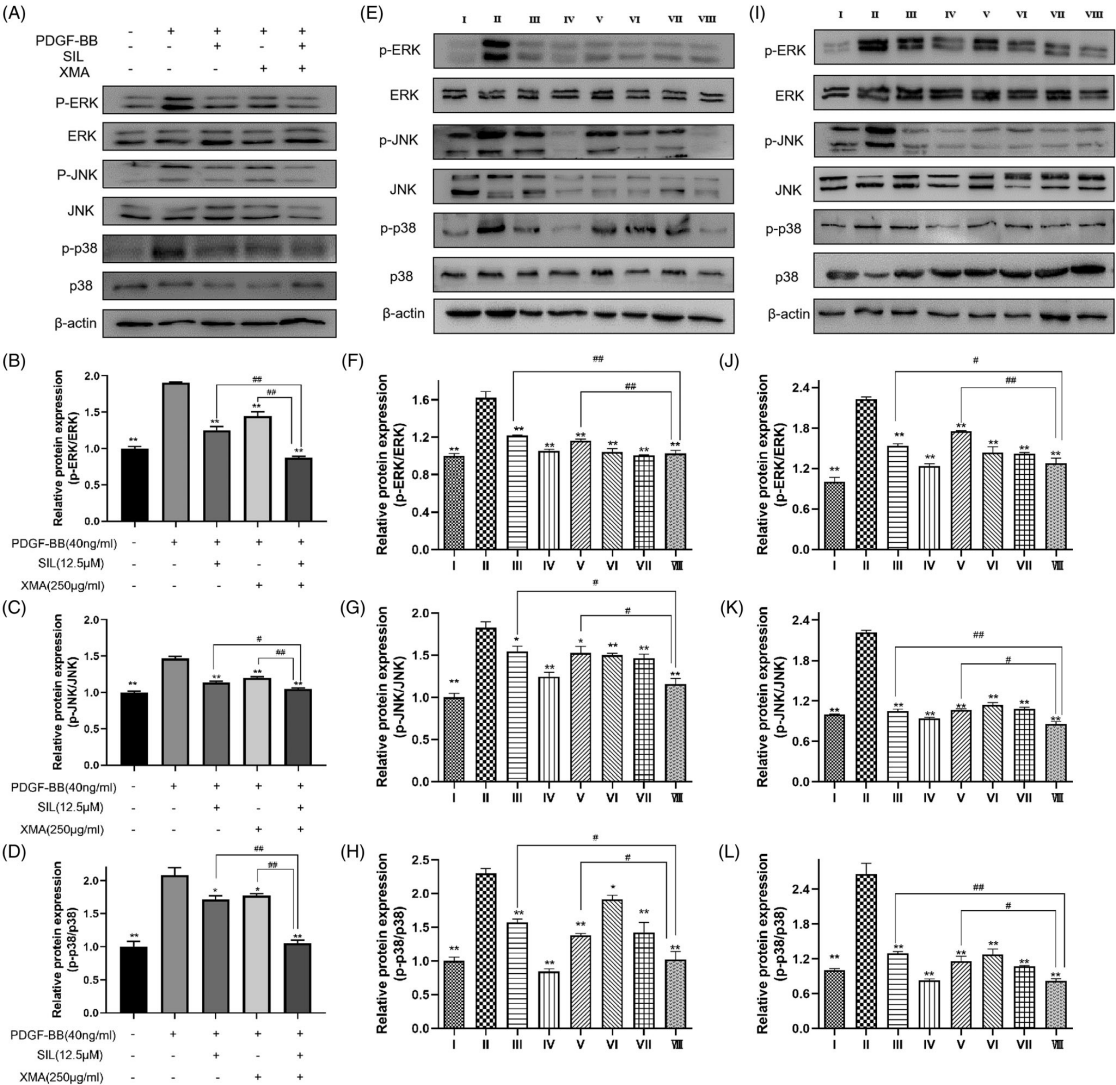
Effects of SIL and XMA administered separately or in combination could inhibit MAPK signalling pathway in vitro and in vivo. (A-D) The expression of p-ERK, ERK, p-JNK, JNK, p-p38MAPK and p38MAPK in PASMCs were assayed by Western blot. The phosphorylated protein bands were standardized to total protein expression bands. Data are presented as the mean ± SEM. *p < 0.05, **p < 0.01 vs. PDGF-BB group; ^#^*p* < 0.05, ^##^*p* < 0.01 vs. SIL+XMA group. (EH) The expression of p-ERK, ERK, p-JNK, JNK, p-p38MAPK and p38MAPK in lung were assayed by Western blot. The phosphorylated protein bands were standardized to total protein expression bands. (I-L) The expression of p-ERK, ERK, p-JNK, JNK, p-p38MAPK and p38MAPK in heart were assayed by Western blot. The phosphorylated protein bands were standardized to total protein expression bands. Group I: control group received saline; Group II: MCT group received MCT(60 mg/kg); Group III: SIL Low group received MCT+SIL at 10 mg/kg/day; Group IV: SIL high group received MCT+SIL at 30 mg/kg/day; Group V: XMA High group received MCT+XMA at 62.9 mg/kg/day; Group VI: SIL(Low)+XMA(Low) group received SIL(10 mg/kg)+XMA at 125.8 mg/kg/day; Group VII: SIL(Low)+XMA(Medium) group received SIL(10 mg/kg)+XMA at 251.6 mg/kg/day; Group VIII: SIL(Low)+XMA(High) group received SIL(10 mg/kg)+XMA at 251.6 mg/kg/day. Data are presented as the mean ± SEM. *p < 0.05,**p < 0.01 vs. Group II; ^#^*p* < 0.05, ^##^*p* < 0.01 vs. Group VIII.

Dysfunction of the MAPK signalling pathway has been observed in various models of pulmonary hypertension (Awad et al. 2016). To clarify whether SIL in combination with XMA can treat pulmonary hypertension by inhibiting MAPK signalling pathway, we detected the protein expression of p-ERK/ERK, p-JNK/JNK and p-p38/p38 *in vitro* and *in vivo*. As indicated in Figure 8(A–D). MAPK signalling pathway was active by 40 ng/mL PDGF-BB stimulation for 24 h. However, SIL (12.5 μΜ) and XMA (250 μg/mL) treatment inhibit the MAPK signalling pathway. Meanwhile, when the cells were exposed to XMA that combined with SIL, MAPK signalling pathway was inhibited. Similarly, the protein expression of p-ERK/ERK, p-JNK/JNK and p-p38/p38 in the lung and heart were inhibited by the treatment of the combination of SIL and XMA (Figure 8(E–L).

## Discussion

Pulmonary hypertension is a chronic cardiopulmonary disease caused by a diversity of genetic and pathogenic causes. It is characterized by dysfunction of pulmonary endothelial cells, progressive growth of PASMCs, and subsequent intimal and neointimal thickening. Typical clinical symptoms include dyspnoea, fatigue, and angina pectoris. Currently, PAH cannot be cured except for lung transplantation. It often requires lifelong medication to improve symptoms and survival (Xu et al. 2018).

Sildenafil is a highly selective phosphodiesterase-5 inhibitor, which can enhance the cGMP pathway, ultimately affect the synthesis and release of vasoactive factor NO, and regulate the level of free Ca^2+^ in cells, resulting in vascular dilatation and reduction of pulmonary artery pressure (Gao et al. 2020). It has been proven that sildenafil alone or in combination with other drugs has a certain effect on various types of pulmonary hypertension, and its price is cheaper than prostacyclin drugs and endothelin receptor antagonists (Rashid et al. 2019). Therefore, its clinical application prospects, especially the effect of combined use with other drugs is compelling.

More and more studies have shown that traditional Chinese medicine is also effective in treating pulmonary hypertension, whether in basic or clinical studies (Dang et al. 2020). At present, basic western medicine combined with traditional Chinese medicine is widely used in the clinical treatment of diseases (Chen et al. 2020). Previous research has revealed that traditional Chinese medicine such as paeoniflorin, ginsenoside Rg_1_, kaempferol and Tanshinone IIA could treat pulmonary hypertension. Xinmai 'an extract includes these four monomers. Therefore, we hypothesized that Xinmai 'an could treat pulmonary hypertension. In addition, in order to reduce the dose of SIL and reduce toxicity, we adopted a multi-drug combination (SIL and XMA) therapy strategy for pulmonary hypertension.

Previous studies have shown that PDGF-BB can promote the proliferation and migration of pulmonary artery smooth muscle cells, aggravating the degree of pulmonary hypertension (Zhao et al. 2014). In our study, in order to demonstrate the effects of SIL and XMA on the activity of pulmonary artery smooth muscle cells, we first examined the effects of these two drugs on cell viability and demonstrated that they dramatically inhibited the proliferation of PASMCs. Our goal was to improve SIL’s efficacy in the treatment of pulmonary hypertension by combining it with XMA. So SIL and XMA were used at minimum effective concentrations. The results showed that XMA could improve SIL’s efficacy in the treatment of PAH by decreasing cell viability more effectively. Therefore, we used these two drug concentrations for subsequent experiments. Meanwhile, migratory growth is another important cause of PAH. MMP 2 and MMP 9 are the protein biomarkers representing cell migration and invasion (Kolli-Bouhafs et al. 2012). In the current study, we found a reduction in cell scratch area and the number of cell migrations after SIL or XMA treatment. MMP2 and MMP9 protein expression were also down-regulated in the combination group compared with the SIL or XMA administered alone group. It is commonly known that proliferation arrest is closely related to cell apoptosis. And, apoptosis exerts an important part in PAH. Therefore, inhibition of pulmonary arterial smooth muscle cell apoptosis is an effective way to treat pulmonary hypertension (Crosswhite and Sun 2014). In the present study, we found that the XMA could improve SIL’s efficacy in the treatment of PAH by increasing the number of apoptotic cells. At the same time, the expression of apoptosis-related proteins increased, while the expression of anti-apoptotic proteins decreased.

Increasing evidence shows the importance of oxidative stress and inflammation in the development of pulmonary hypertension (Fessel and West 2015). The animal model of PAH showed increased oxidative stress and inflammation, both of which promote PASMC proliferation, pulmonary vascular remodelling and right ventricular failure (Archer et al. 2010). In the present study, we found that ROS generation, MDA content and the level of inflammatory factors such as IL-1β, IL-6, TNF-α were down-regulated in the combination group compared to SIL or XMA administered alone group. The results suggested XMA could improve SIL’s efficacy in the treatment of PAH by reducing ROS generation, MDA content and the level of inflammatory factors.

In order to further study the therapeutic effect of drugs on pulmonary hypertension, we established the rat model of monocrotaline-induced PAH. The results showed that the two drugs reduced RVSP, RV remodelling and pulmonary arterial remodelling, which was decreased significantly in the co-administration group, and the result of the SIL (Low)+XMA (High) group was similar to the level of high dose of sildenafil. These results suggest that XMA can treat pulmonary hypertension to some extent and XMA might be used as a potential co-administered drug for increasing sensitivity and efficacy of SIL.

**Figure 9.**
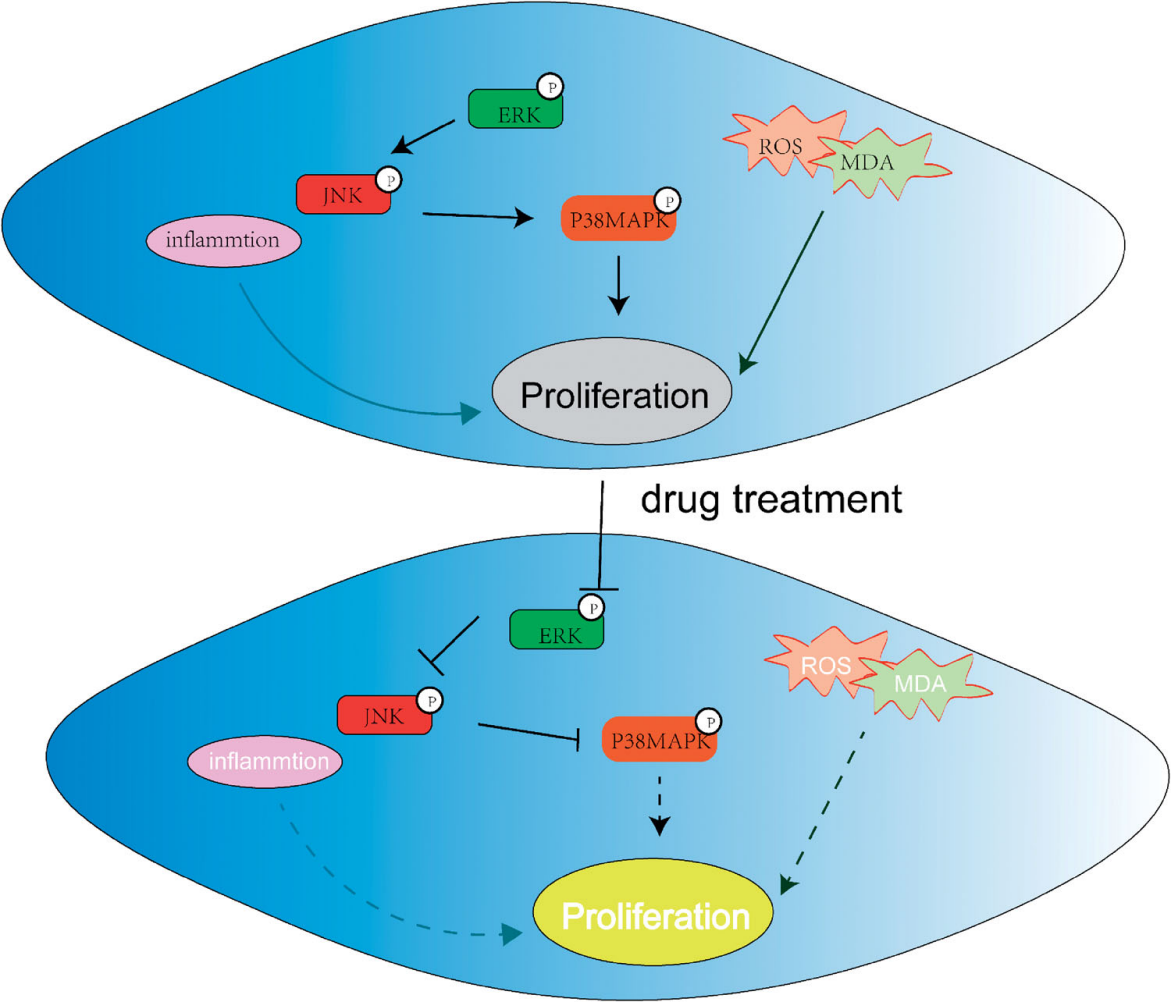
Graphical abstract of how XMA and SIL reduced pulmonary hypertension via the MAPK signalling pathway.

Finally, we investigated the molecular mechanisms of the effects of SIL or XMA on pulmonary hypertension *in vivo* and *in vitro*. Increasing pieces of evidence indicated that activation of MAPK signalling pathway in MCT-induced models is the cause of PAH pathology (Sluiter et al. 2012). Besides, sildenafil inhibited the phosphorylation of ERK to inhibit PASMCs proliferation (Tantini et al. 2005). The antiproliferative effects of XMA’s monomers such as ginsenoside Rg_3_ were largely associated with deregulating the MAPK signalling pathway (Cheng and Xing 2019). In the present study, we demonstrated that SIL or XMA could block MAPK signalling pathway activation induced by the stimulation of PDGF-BB or MCT. And MAPK signalling was also down-regulated in the combination group more obviously compared with the SIL or XMA administered alone group. Therefore, XMA can improve the therapeutic effect of SIL for pulmonary hypertension by further reducing inflammatory activation, reducing oxidative stress, and inhibiting MAPK signalling pathway, as shown in Figure 9.

## Conclusions

In conclusion, our results indicate that XMA could inhibit the proliferation of PASMCs *in vitro*, and reduce pulmonary hypertension in MCT-induced rats. The combination of SIL and XMA effectively improved hemodynamics and suppressed PASMCs growth, which can reduce the dose of sildenafil and have a better therapeutic effect. This study may provide a theoretical basis for the clinical treatment of pulmonary hypertension.
